# Increased Reticulocytosis during Infancy Is Associated with Increased Hospitalizations in Sickle Cell Anemia Patients during the First Three Years of Life

**DOI:** 10.1371/journal.pone.0070794

**Published:** 2013-08-07

**Authors:** Emily Riehm Meier, Colleen Byrnes, Y. Terry Lee, Elizabeth C. Wright, Alan N. Schechter, Naomi L. C. Luban, Jeffery L. Miller

**Affiliations:** 1 Molecular Medicine Branch, National Institute of Diabetes and Digestive and Kidney Diseases, National Institutes of Health, Bethesda, Maryland, United States of America; 2 Center for Cancer and Blood Disorders, Children’s National Medical Center, Washington, District of Columbia, United States of America; 3 Office of the Director, National Institutes of Diabetes and Digestive and Kidney Diseases, National Institutes of Health, Bethesda, Maryland, United States of America; 4 Department of Pediatrics, The George Washington University Medical Center, Washington, District of Columbia, United States of America; Emory University/Georgia Insititute of Technology, United States of America

## Abstract

**Objective:**

Among older children with sickle cell anemia, leukocyte counts, hemoglobin, and reticulocytosis have previously been suggested as disease severity markers. Here we explored whether these blood parameters may be useful to predict early childhood disease severity when tested in early infancy, defined as postnatal ages 60–180 days.

**Study Design:**

Data from fifty-nine subjects who were followed at Children’s National Medical Center’s Sickle Cell Program for at least three years was retrospectively analyzed. Comparisons were made between white blood cell counts, hemoglobin and reticulocyte levels measured at ages 60–180 days and the clinical course of sickle cell anemia during infancy and childhood.

**Results:**

A majority of subjects had demonstrable anemia with increased reticulocytosis. Only increased absolute reticulocyte levels during early infancy were associated with a significant increase in hospitalization during the first three years of life. Higher absolute reticulocyte counts were also associated with a markedly shorter time to first hospitalizations and a four-fold higher cumulative frequency of clinical manifestations over the first three years of life. No significant increase in white blood cell counts was identified among the infant subjects.

**Conclusions:**

These data suggest that during early infancy, increased reticulocytosis among asymptomatic SCA subjects is associated with increased severity of disease in childhood.

## Introduction

Although the genetic basis of sickle cell anemia (HbSS, SCA) is well understood, the clinical phenotype is highly variable and difficult to predict. [1] Evidence of increased hemolysis begins during the first year of life as sickle hemoglobin (HbS) replaces fetal hemoglobin (HbF; α_2_γ_2_). With time, the consequences of intracellular HbS polymerization lead to susceptibility to infections, pain episodes, vascular and multi-organ damage, and shortened life expectancy. Splenic dysfunction affects over 90% of subjects before adulthood, [2] therefore antibiotic prophylaxis [3] is used during infancy to reduce the incidence of life threatening infection. [4] Patients are also treated with hydroxyurea (HU), chronic transfusions, and bone marrow transplantation, all of which have been shown to improve certain disease manifestations in children and adults. Monthly blood transfusions have decreased the stroke risk in SCA subpopulations. [5] However, because all of these therapies have significant morbidity, caution is appropriately applied with any such treatment decisions. [6]–[8] Ongoing efforts to identify infants and children at high risk for severe disease are especially relevant for choosing treatment options.

Ideally, SCA subjects at risk for severe disease should be identified as complications develop to better target therapeutic choices and timing. SCA researchers are thus challenged to predict disease severity before the disease is fully manifested. In the United States, hemoglobinopathy screening programs permit genetic diagnosis in newborns with SCA prior to the onset of any disease manifestations. While approximately 3,000 infants are born in the US with SCA annually, it is estimated that 250,000 infants are born annually with SCA in Sub-Saharan Africa. [9] Newborn screening programs are being implemented in select African countries that will permit early institution of supportive care measures including penicillin prophylaxis. [10] With newborn screening of infants becoming more widespread in Africa, disease severity markers that are useful at an early age are a priority for translational and clinical research. In resource-poor countries, efforts to target therapy to those children who are at highest risk of disease complications are especially important. Studies of genetic markers for the clinical phenotype are advancing, [11] but clinical markers that are strongly predictive for individual infants with SCA have not yet been identified.

Previously, a large prospective cohort study was utilized for identifying disease severity predictors in children with sickle cell disease. [12]–[15] However, the severity predictors identified in that cohort were not fully validated in a subsequent analysis. [16] The goal of our study is the identification of a globally available SCA disease severity predictor that is amenable to testing in infants prior to manifestation of the disease. We sought an inexpensive test that may be applied to identify infants at higher risk for severe disease during early childhood.

## Materials and Methods

### Ethics Statement

Approval for the research protocol and consent documents pertaining to this study was granted by the Intramural National Institute of Diabetes and Digestive and Kidney Diseases and the Children’s National Medical Center Institutional Review Boards. Written consent from parents and guardians for participants less than age 18 years and assent, when applicable, were obtained prior to enrollment.

### Subject Eligibility and Enrollment

Retrospective chart review was performed on a subset of HbSS patients who were part of a longitudinal, observational cohort study to identify markers of SCA severity. This cohort consists of a convenience sample of pediatric sickle cell anemia (SCA) patients through age 21 years who received care at the Children’s National Medical Center (CNMC) Sickle Cell Program. Only those study participants who had a steady state complete blood count and reticulocyte count during early infancy (between the ages of 60 and 180 days) and were born before January 1, 2010 (providing at least 3 years of follow-up) are included in this report. Reticulocyte number and hemoglobin level (Hb) were measured using a Sysmex XE 2100 hematology analyzer (Sysmex America, Mundelein, IL).

### Analysis of Clinical Outcome Variables

Study outcomes were classified as follows:

1. Painful vaso-occlusive events requiring hospitalization and intravenous (iv) narcotic pain medication (VOC).
2. Acute chest syndrome (ACS) defined as infiltrate on chest Xray (CXR) accompanied by three or more of the following: fever (temperature ≥38.5 degrees Celsius), cough, wheezing, chest pain, or tachypnea. [17]
3. Splenic sequestration defined as: splenic enlargement accompanied by a hemoglobin 2 g/dL below baseline with evidence of bone marrow compensation (increased absolute reticulocyte count). [18]

If subjects had more than one SCA-associated complication during the first hospitalization, the chief complaint at hospital presentation (e.g. leg pain or cough) was used as the cause for the first hospitalization.

### Statistical Methods

Statistical analyses were performed using SAS® version 9.2. A t-test was used to compare the absolute reticulocyte count and hemoglobin of hospitalized and non-hospitalized infants. The four hospitalization groups (No Hospitalization, ACS, splenic sequestration and VOC) were compared using an analysis of variance with Dunnett’s adjustment for multiple comparisons. Logistic regression was used to evaluate the relationships between absolute reticulocyte count and hemoglobin and hospitalization. A Receiver Operator Characteristic (ROC) curve was generated using logistic regression in order to compare the sensitivity (true positive rate) and 1-specificity (false positive rate) for absolute reticulocyte counts versus hemoglobin levels as predictors for probability of hospitalization. Subjects were divided into four groups using cut-offs suggested by the ROC curve. A chi-square test for trend was used to compare the hospitalization rates in the four groups. For other analyses the groups were analyzed as a categorical variable rather than a continuous variable. The Kaplan Meier method was used to estimate the time to first hospitalization and Cox regression was used to estimate the hazard ratio for subjects in these four groups. The event rate per person-years in the first 3 years was calculated by dividing the total number of events by the total years of follow-up up to 3 years and tested using Poisson regression. Cox regression for repeated events was used to estimate the cumulative number of hospitalizations in the first three years for these same four groups. Data are presented as mean ± standard deviation (SD) unless otherwise indicated. A p value of <0.05 was considered statistically significant.

## Results

### Analysis of SCA Severity Markers During Early Infancy

**Figure 1:**
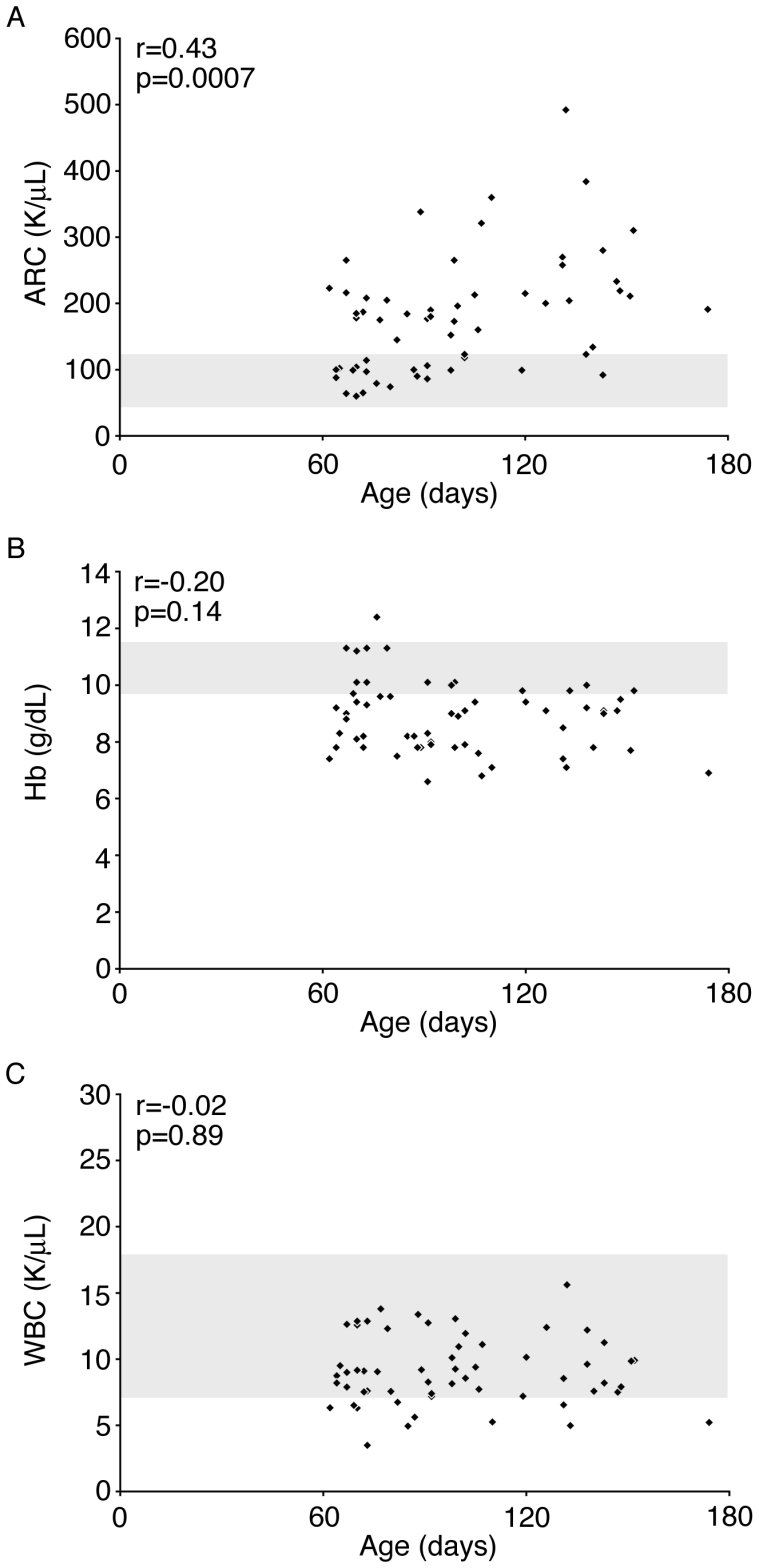
Absolute Reticulocyte Counts, Hemoglobin and White Blood Cell distribution during early infancy in a contemporary, observational SCA cohort. A. Distribution of absolute reticulocyte count values among 59 subjects ages 60–180 days. B–C. Hemoglobin and White blood cell count distribution in the same cohort. Shaded area represents the normal range for a 3-month-old healthy infant. [19] Age in days is shown on the x-axis in each panel. Correlation coefficients (r) and p- values are shown in each panel. ARC, Absolute Reticulocyte Count; Hb, hemoglobin; WBC, White Blood Cell count.

Fifty-nine subjects who were born before January 1, 2010 had a steady state CBC (including absolute reticulocyte count) between the ages of 60 and 180 days. Previous analyses [13]–[15] identified white blood cell count, hemoglobin, and reticulocytosis in young children as blood parameters that may be useful for prediction of future disease severity. Dactylitis was also identified as a disease severity marker in those studies. In order to determine if these markers were useful earlier in life, before six months of age, we compared peripheral blood white blood cell counts, hemoglobin concentrations, and absolute reticulocyte counts among the 59 patients with those levels measured in healthy infants. [19] None of the patients in our cohort had dactylitis before the age of 6 months, so this marker was not investigated further. Consistent with previous reports, the mean absolute reticulocyte count in this group of SCA infants was 179±89 K/uL. As shown in Figure 1A, there was an upward trend of absolute reticulocyte count in the older infants, and the absolute reticulocyte count values were mostly distributed between 100–300 K/uL. Conversely, anemia (Hb <9.5) occurred before age 6 months in over two-thirds (69.5%) of our cohort (Figure 1B). The mean hemoglobin for the cohort was 8.9±1.3 g/dL. None of the patients had an elevated white blood cell count (Figure 1C).

### Absolute Reticulocyte Count, Hemoglobin, White Blood Cell Count and SCA Clinical Events

**Figure 2:**
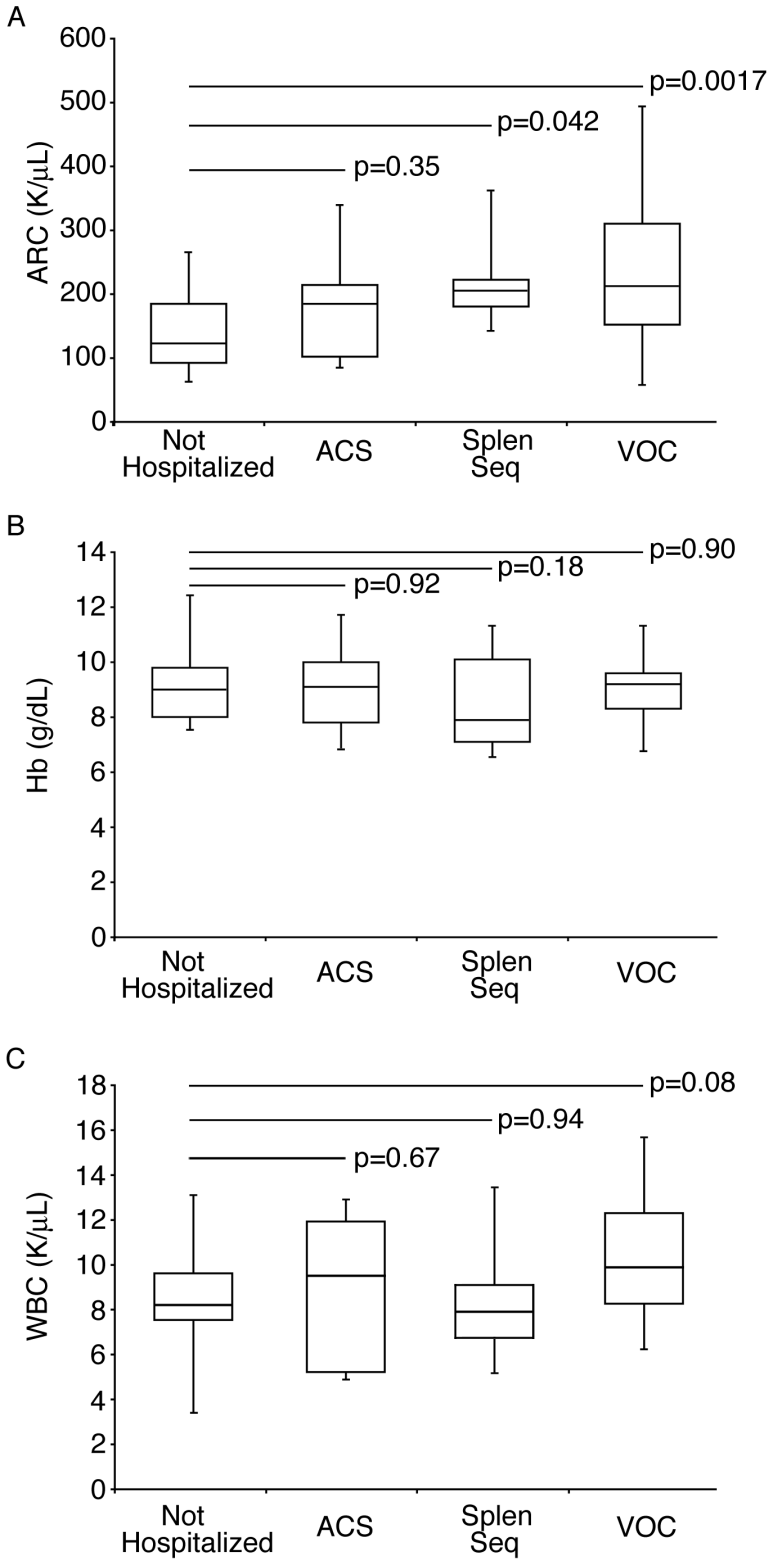
Comparison of absolute reticulocyte counts and blood hemoglobin and white blood cell levels according to early hospitalization. A. Steady state absolute reticulocyte count values measured during early infancy (60–180 days; shown in box and whiskers format) for subjects with no history of hospitalization (No Hosp., n = 23) versus subjects who required early hospitalization (before age three years) for each cause of hospitalization [acute chest syndrome (ACS, n = 11), splenic sequestration (Splen seq, n = 10) and vaso-occlusive crisis (VOC, n = 15)]. Equivalent analyses were performed using hemoglobin levels (Panel B) and white blood cell counts (Panel C). Unadjusted significance (p values) of differences from the non-hospitalized group is shown on top of the bars. After adjusting for multiple comparisons, the absolute reticulocyte count for the VOC group remained significantly higher than that for the group with no hospitalizations. ARC, Absolute Reticulocyte Count; Hb, hemoglobin; WBC, White Blood Cell count.

For this study, hospitalization (or lack thereof) by three years of age was defined and studied as being indicative of early disease severity in these 59 subjects. The mean age at first hospitalization was 17.6±8.3 months for those subjects who required a hospitalization in the first 3 years of life. Absolute reticulocyte counts, hemoglobin levels, and white blood cell counts were explored for their correlation with hospitalization in the first three years of life. The mean absolute reticulocyte count level (140±63 K/uL) for the 23 subjects who were not hospitalized was significantly lower than that of the remaining 36 subjects who required hospitalization during the first three years of life (204±94 K/uL, p = 0.0054), while the mean hemoglobin was not different between the two groups (Figure 2A, No Hospitalization 9.0±1.1 g/dL vs. Hospitalized group 8.8±1.4 g/dL, p = 0.53). The absolute reticulocyte counts of 11 subjects who were first hospitalized for ACS (mean absolute reticulocyte count 168±76 K/uL) was not significantly different than those who were not hospitalized (p = 0.35). In contrast, the 10 subjects who were first hospitalized for splenic sequestration and the 15 subjects first hospitalized for VOC had significantly higher absolute reticulocyte counts than those who were not hospitalized (205±72 K/uL and 230±114 K/uL respectively, p = 0.042 and 0.0017). After adjusting for multiple comparisons, the absolute reticulocyte count for the VOC group remained significantly higher than that for the group with no hospitalizations (p<0.05). As shown in Figure 2B, the hemoglobin levels of the subjects first hospitalized for ACS (9.0±1.3 g/dL), splenic sequestration (8.4±1.7 g/dL) and VOC (8.9±1.2 g/dL) were not significantly different than those subjects who did not require hospitalization (p = 0.92, 0.18, and 0.90). White blood cell counts (Figure 2C) were not significantly different in those patients who required hospitalization when compared to those who did not. [ACS (mean white blood cell count 9.0+3.0 K/uL), splenic sequestration (mean white blood cell count 8.6+2.6 K/uL), VOC (mean white blood cell count 10.2+2.7 K/uL), no hospitalization (mean white blood cell count 8.6+2.3 K/uL), p = 0.67, 0.97, 0.08, respectively].

**Figure 3:**
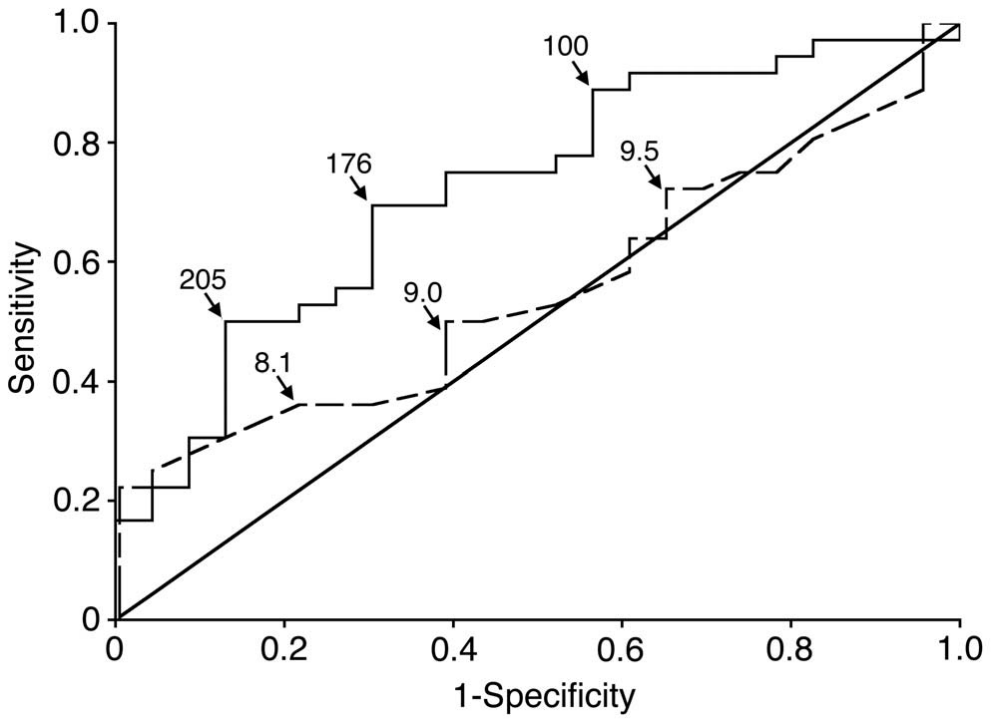
Receiver operator characteristic (ROC) curve analysis. ROC curve comparing the patients who required or did not require hospitalization within the first 3 years of life. Absolute reticulocyte count (solid line) hemoglobin level (dashed line) values at selected points (arrows) along the ROC are included. Sensitivity is shown on the y-axis, and 1-specificity is listed on the x-axis. p values represent significance of area under the curve. ARC, Absolute Reticulocyte Count; Hb, hemoglobin; AUC, area under the curve with 95% confidence intervals (CI).

Figure 3 shows the probability of hospitalization versus no hospitalization as illustrated by ROC curve and Area Under the Curve (AUC) analyses for absolute reticulocyte count and hemoglobin. The odds ratios from the logistic regression used to construct the ROC were 1.74 (95% CI 1.14–2.65) for each 50-unit change in absolute reticulocyte count and 0.87 (95% CI 0.57–1.33) for each unit change in Hb. The AUC (0.72; 95% CI 0.59–0.86; p = 0.0097) demonstrates that increasing reticulocytosis (solid line) is associated with a significantly higher risk of hospitalization before age 3 years. An AUC of 0.5 indicates that this discriminator (absolute reticulocyte count in this instance) is no greater than chance, while an AUC of 1.0 means it is a perfect discriminator with 0 false positives and 0 false negatives. [20] All 6 patients who had an absolute reticulocyte count greater than 280 K/uL were hospitalized within the first 3 years of life (100% specificity) while the one patient with an absolute reticulocyte count less than 64 K/uL was not hospitalized in the first three years of life (100% sensitivity). The AUC for hemoglobin (0.55, 95% CI 0.41–0.70; p = 0.52) revealed that hemoglobin was not useful for predicting hospitalizations in this cohort. Although the AUC for absolute reticulocyte count (0.72) was higher than the AUC for hemoglobin (0.55), this difference was not statistically significant (p = 0.12).

### Time to First Event and Cumulative Number of Events

**Figure 4:**
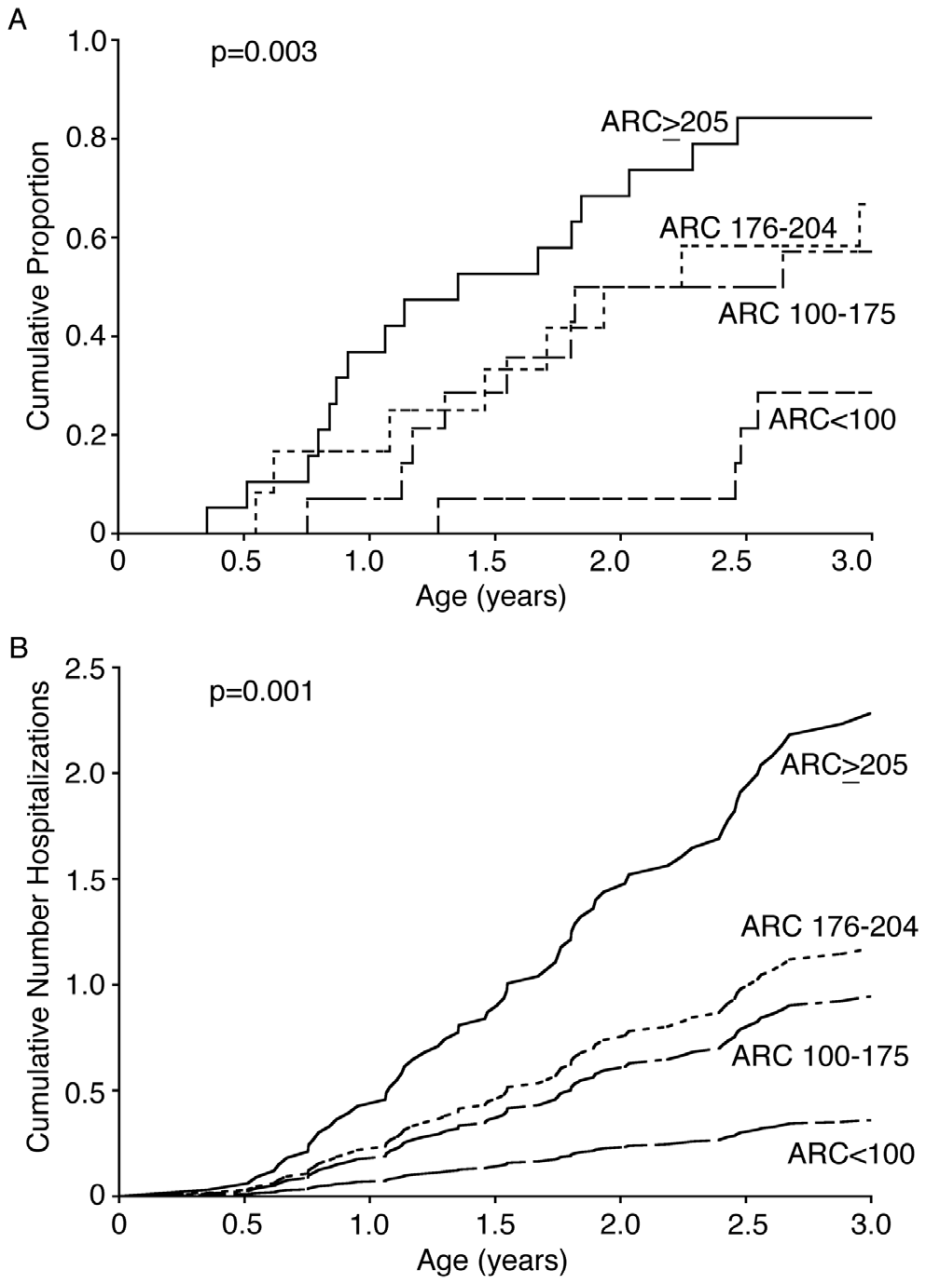
Time to first event and cumulative mean frequency of events. A. Kaplan Meier curve showing the cumulative proportion of events by patient groups. The groups were divided by absolute reticulocyte count based on the ROC curve seen in Figure 3 [absolute reticulocyte count >205 K/uL (solid line, n = 19, 0.84, 95% CI 0.68–1.01), absolute reticulocyte count between 176 and 204 K/uL (dotted line, n = 12, 0.67, 95% CI 0.40–0.93), absolute reticulocyte count between 100 and 175 K/uL (dashed & dotted line, n = 14, 0.57, 95% CI 0.31–0.83), absolute reticulocyte count <100 K/uL (dashed line, n = 14, 0.29, 95% CI 0.05–0.52)]. B. Cumulative mean frequency of events curves (cumulative frequency of hospitalizations on the y-axis vs. age in years on the x-axis) for the groups described in panel A [absolute reticulocyte count >205 (cumulative mean frequency of events 2.28, 95% CI 1.49–3.49), absolute reticulocyte count 176–204 (cumulative mean frequency of events 1.17, 95% CI 0.67–2.04), absolute reticulocyte count 100–175 (cumulative mean frequency of events 0.94, 95% CI 0.51–1.75), absolute reticulocyte count <100 (cumulative mean frequency of events 0.36, 95% CI 0.15–0.88)]. In both panels, the p value represents the differences among the four groups. ARC, Absolute Reticulocyte Count.

The time to first hospitalization and the number of hospitalizations by age three years were next used as markers of increased disease severity. The infants were grouped according to their absolute reticulocyte counts from 60–180 days using the ROC data shown in Figure 3: absolute reticulocyte count less than 100 K/uL (group 1), absolute reticulocyte count between 100 and 175 K/uL (group 2), absolute reticulocyte count between 176 and 204 K/uL (group 3) and absolute reticulocyte count greater than or equal to 205 K/uL (group 4). Twenty-nine percent (4/14) of patients in group 1, 57% (8/14) in group 2, 67% (8/12) in group 3, and 84% (16/19) in group 4 had an event by age 3 years (p = 0.0014). We first compared the time to first hospitalization for these four groups using the Kaplan-Meier method and Cox Regression. Kaplan-Meier estimates of the time to first hospitalization are shown in Figure 4A. The Cox regression hazard ratios, compared to group 1, were 2.76 (95% CI 0.83–9.20, p = 0.098) for group 2, 3.45 (95% CI 1.04–11.49, p  = 0.044) for group 3, and 6.28 (95% CI 2.08–19.00, p = 0.0011) for group 4.

The total number of clinical events was calculated for each group and cumulative frequency of events was compared. The number of events per patient year was increased with increasing absolute reticulocyte count (5 events, 0.12 events/patient years for group 1, 12 events (0.31) for group 2, 12 events (0.38) for group 3, and 41 events (0.81) for group 4, p = <0.0001). Before three years of age, the average cumulative number of events was 0.23 for group 1, 0.60 for group 2, 0.74 for group 3, and 1.44 for group 4, p  = 0.0010 for a Cox regression analysis of recurrent events (Figure 4B).

## Discussion

Data from a previously reported cohort of children with sickle cell disease identified baseline hemoglobin, white blood cell count and early dactylitis as predictors of severe SCA. [15] Increasing reticulocytosis was also associated with a more severe SCA course. Here we report data collected during early infancy from 59 subjects with the goal of identifying markers of disease severity during the first three years of life. None of the subjects presented with dactylitis or leukocytosis before 6 months of age. However, they manifested more significant anemia and reticulocytosis as compared to healthy children. Thus, levels of anemia and reticulocytosis were further analyzed to determine if either could be used as a very early marker of SCA severity that may be measured prior to the onset of disease sequelae. The switch from predominantly fetal to primarily sickle hemoglobin during the first several months of life leads to anemia with reticulocytosis as a nearly universal feature of the disease. In the healthy infants, recovery from the so-called “physiologic anemia” [21] involves the production of erythrocytes with almost entirely HbA. In infants with SCA, the erythrocytes containing high levels of HbF circulating at birth are replaced by erythrocytes with high levels of HbS and decreased levels of HbF. [22] Once the remaining fetal cells are cleared from the circulation, anemia develops. [23] The subsequent reduction in hematocrit leads to increased production of HbS-containing cells, and tissue hypoxia appears to remain as a central driver of continued increased reticulocyte production. A more robust reticulocyte response is predicted in those SCA infants who are less able to maintain HbF production, since the physiologic nadir occurs during the fetal-to-adult hemoglobin transition during human ontogeny. In the absence of signs or symptoms of clinical disease, the infants identified here as having increased reticulocyte counts of greater than or equal to 205 K/uL before the age of 180 days were found to have a significantly increased risk of hospitalization for sickle cell-associated sequelae before age three years.

The cohort study presented here was limited by its small sample size and retrospective design, which prevented infants from being evaluated at standard time points. Additionally, HbF data was not available for all infants between the ages of 2 and 6 months, which limited comparison of HbF levels to the SCA severity. None of the enrolled patients in the contemporary cohort died or had a stroke, arguably the two most significant consequences of SCA. When tested in older children, reticulocytosis has also been reported to be a risk factor for cerebrovascular disease. [24] With improvement in the supportive care of pediatric SCA patients, death and stroke are rare events in early childhood. [25] Despite the recognition that all SCA infants undergo a transition from health to chronic disease during infancy, there exists a paucity of data concerning the clinical status of infants during this transition period. No prior studies identified a predictive marker of sickle cell disease severity during this early stage of life. The absolute reticulocyte count is an inexpensive laboratory assay that is currently available and amenable for clinical assessment of young infants by the global community. Our data suggest that quantifying absolute reticulocyte counts during early infancy may be useful for predicting disease severity when the disease is initially manifested in childhood. However, the limitations of this study suggest that clinical use of absolute reticulocyte count in early infancy as a predictor of hospitalizations in the first three years of life should be currently viewed as tentative until validation is achieved using larger, more socio-economically and geographically diverse cohorts of patients.

The findings highlight the importance of newborn screening programs for infants with SCA since the disease phenotype is usually delayed in infants and children until after the age of six months. Further study is needed to determine the potential for infant reticulocytosis to predict disease severity among older children or adults, and prospective examinations of reticulocytosis may explore discrete cohorts including subjects being considered for HU, transfusion, or transplantation. Since the magnitude of HbF loss is variable between infants [22], [26], detailed studies are needed to determine if greater loss of HbF during infancy correlates with higher levels of reticulocytosis. The role of HbF levels in identifying high-risk infants may also be limited in developing countries because of difficulties in obtaining these results, while absolute reticulocyte counts are more readily obtainable. Since the patient cohort studied here receive their care from a large clinical center in the United States, ARC from patient cohorts receiving care in other global locales, including Africa, should be studied for comparison. Future research should also include detailed studies of the sickle cell infants’ reticulocyte biology, hemolysis (bilirubin and LDH levels), splenic function, and genomics in order to better understand the initial manifestation of the disease. While sickle hemoglobin polymerization remains the incipient feature of SCA, [27] the host’s erythropoietic response, including the production and release of reticulocytes from the marrow that are more adherent to the vascular endothelium, may play a significant role in determining the disease phenotype. [28].
